# Real-time PCR Demonstrates High Prevalence of *Schistosoma japonicum* in the Philippines: Implications for Surveillance and Control

**DOI:** 10.1371/journal.pntd.0003483

**Published:** 2015-01-21

**Authors:** Catherine. A. Gordon, Luz P. Acosta, Geoffrey N. Gobert, Remigio M. Olveda, Allen G. Ross, Gail M. Williams, Darren J. Gray, Donald Harn, Yuesheng Li, Donald P. McManus

**Affiliations:** 1 Molecular Parasitology Laboratory, Infectious Diseases Division, QIMR Berghofer Medical Research Institute, Brisbane, Australia; 2 Discipline of Epidemiology and Biostatistics, School of Population Health, University of Queensland, Brisbane, Australia; 3 Department of Immunology, Research Institute of Tropical Medicine, Manila, Philippines; 4 Griffith Health Institute, Griffith University, Queensland, Australia; 5 Research School of Population Health, College of Medicine, Biology and Environment, the Australian National University, Canberra, Australia; 6 University of Georgia, College of Veterinary Medicine, Athens, Georgia, United States of America; Swiss Tropical and Public Health Institute, SWITZERLAND

## Abstract

**Background:**

The Philippines has a population of approximately 103 million people, of which 6.7 million live in schistosomiasis-endemic areas with 1.8 million people being at risk of infection with *Schistosoma japonicum*. Although the country-wide prevalence of schistosomiasis japonica in the Philippines is relatively low, the prevalence of schistosomiasis can be high, approaching 65% in some endemic areas. Of the currently available microscopy-based diagnostic techniques for detecting schistosome infections in the Philippines and elsewhere, most exhibit varying diagnostic performances, with the Kato-Katz (KK) method having particularly poor sensitivity for detecting low intensity infections. This suggests that the actual prevalence of schistosomiasis japonica may be much higher than previous reports have indicated.

**Methodology/Principal Findings:**

Six barangay (villages) were selected to determine the prevalence of *S. japonicum* in humans in the municipality of Palapag, Northern Samar. Fecal samples were collected from 560 humans and examined by the KK method and a validated real-time PCR (qPCR) assay. A high *S. japonicum* prevalence (90.2%) was revealed using qPCR whereas the KK method indicated a lower prevalence (22.9%). The geometric mean eggs per gram (GMEPG) determined by the qPCR was 36.5 and 11.5 by the KK. These results, particularly those obtained by the qPCR, indicate that the prevalence of schistosomiasis in this region of the Philippines is much higher than historically reported.

**Conclusions/Significance:**

Despite being more expensive, qPCR can complement the KK procedure, particularly for surveillance and monitoring of areas where extensive schistosomiasis control has led to low prevalence and intensity infections and where schistosomiasis elimination is on the horizon, as for example in southern China.

## Author Summary

Schistosomiasis is caused by infection with trematode blood flukes of the genus *Schistosoma. Schistosoma japonicum* is the causative agent of schistosomiasis in the Philippines, China and parts of Indonesia. In the Philippines, 6.7 million people live in endemic areas and 1.8 million are at risk of infection whereas concerted control efforts over the past 50 years in China have reduced the number of infected individuals considerably. Currently used microscopic techniques for diagnosis, notably the Kato-Katz (KK) technique, lack sensitivity in areas with low intensity schistosome infections. We have used a molecular diagnostic approach (qPCR), to assess the prevalence of *S. japonicum* in humans from six barangays in Northern Samar, the Philippines. The qPCR performed considerably better than the KK as a diagnostic procedure and could be an important tool in the future for surveillance and monitoring of areas where extensive schistosomiasis control has led to low prevalence and intensity infections and where schistosomiasis elimination is possible.

## Introduction

*Schistosoma japonicum* is the causative agent of intestinal schistosomiasis in the Philippines, China and parts of Indonesia. In the Philippines, 10 out of 16 regions have reported cases of schistosomiasis, with an estimated 6.7 million people living in endemic areas [1]. Of these, 1.8 million, nearly 2% of the total Philippine population (105 million) [2], are considered to be directly exposed to infection through farming, fishing and other essential occupational and domestic activities involving regular water contact [3,4]. The spread of schistosomiasis japonica requires the presence of the intermediate aquatic snail host, *Oncomelania hupensis quadrasi*, appropriate mammalian definitive hosts and specific environmental conditions to support transmission. Schistosomiasis is a focal disease and in some Filipino communities the prevalence of infection has been reported to be as high as 65% [5–9].

Common diagnostic techniques, such as the microscopy-based Kato-Katz (KK) method, have been shown to have low sensitivity for detecting schistosome infections [10–15]. The low sensitivity of KK means that low intensity infections are missed and individuals harboring schistosomiasis may not be treated, thereby contributing to ongoing transmission that may result in re-emergence in areas thought to be clear of schistosomiasis [16]. In China, for example, after decades of mass drug administration (MDA) with praziquantel (PZQ) and other integrated control measures, the intensity of *S. japonicum* infection is now quite low in many endemic locations, and therefore the parasite is likely to be missed using KK. It has been shown in China that, after successful control, with significant reduction in prevalence and intensity, if control efforts lapse, there can be re-emergence in transmission and infection [16]. This may be due in part to the KK procedure missing low intensity infections and a more sensitive approach, such as the use of molecular diagnostics, may be required to determine whether, for a particular area, elimination has been achieved. Molecular methods are playing an increasingly important role in schistosome diagnosis and identification due to their increased performance when compared with traditional copro-parasitological techniques [13,17–19]. A number of molecular techniques have been used for the detection of schistosome infections including conventional PCR (cPCR), real-time PCR (qPCR) and loop-mediated isothermal amplification (LAMP) [20–25].

We have previously reported a pilot study examining 50 humans for the presence of *S. japonicum* infections in Western Samar, the Philippines, comparing the KK, cPCR and qPCR methods which showed 30.8%, 75.0% and 92.3% prevalence, respectively [26]. Here we substantially expand the scope of the earlier findings, to further assess the prevalence of *S. japonicum* in humans in Samar Province; and evaluate on a larger scale the diagnostic sensitivity of qPCR compared to KK. We highlight the need for more sensitive diagnostic procedures, particularly in areas where the intensity of infection is low, and discuss the possible application of qPCR in current control efforts

## Materials and Methods

### Ethics

Informed written consent was received from all human participants in the study and ethical approval was provided by the Ethics Committee of the Research Institute of Tropical Medicine (RITM), Manila, and the Queensland Institute of Medical Research (QIMR) Human Research Ethics Committee (Approval Number: H0309-058 (P524)).

Meetings were held in each barangay prior to the start of the study to inform the population about the research, and to answer any questions on the procedures to be carried out. Local medical staff then visited each household over the subsequent week with a consent form (signed by all participants or by a parent or legal guardian for minors before the start of the study) for each participant as well as household and individual questionnaire forms, and to further explain the study to individuals. Individual questionnaires (comprising information on their demographics; age, gender and barangay) were completed by each household member, or by the head of the household in the case of minors. Individual identification numbers (IDN) were assigned from the individual questionnaires. Study participants were aged between 4 and 82 years of age.

Participants who were positive by KK for *S. japonicum* were treated with PZQ using the World Health Organisation (WHO) recommended clinical dosage (40 mg/Kg). Local and government health officials were informed of those subjects positive for *S. japonicum* by qPCR so that follow up treatment could be provided.

### Study design

**Figure 1:**
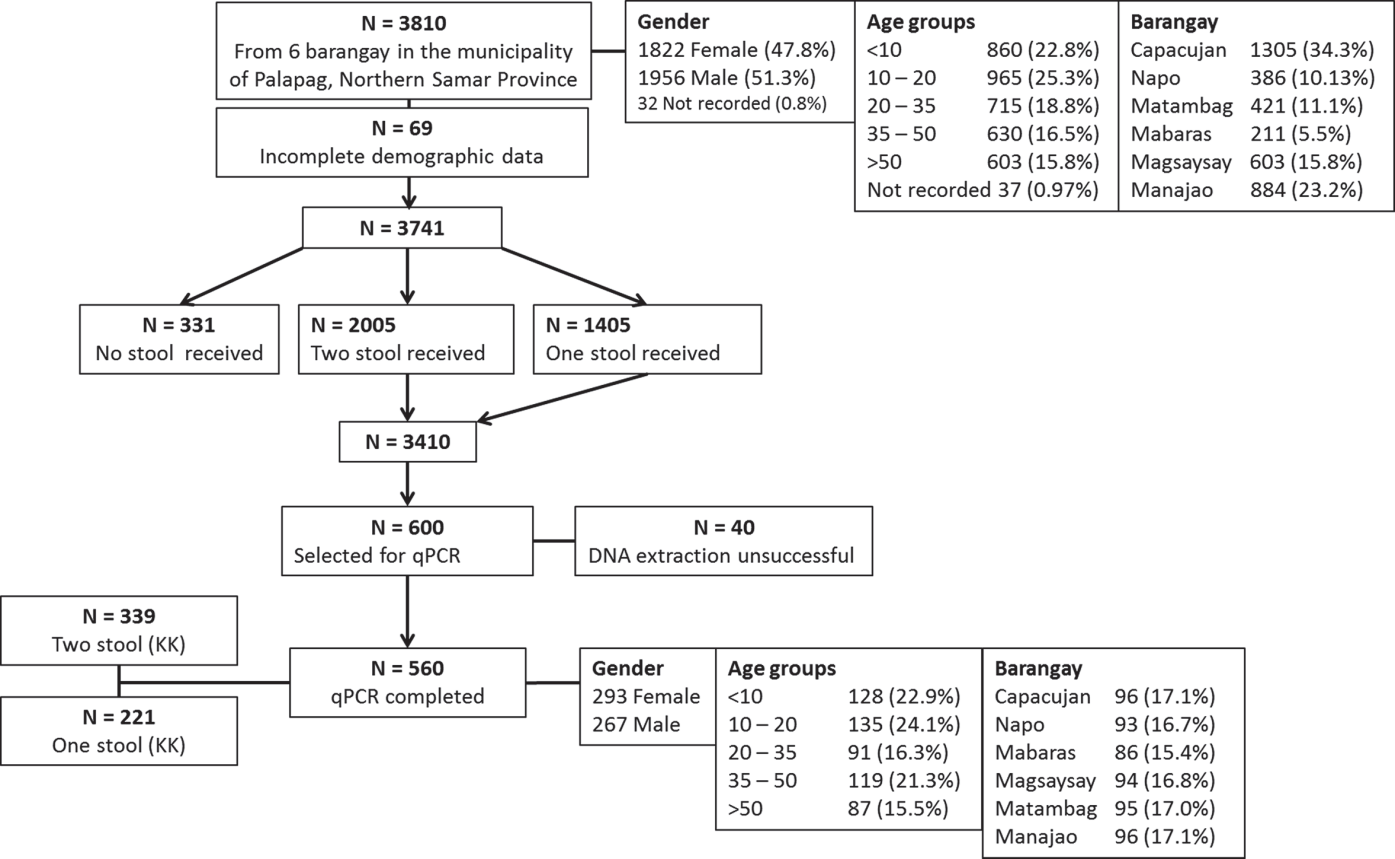
Study profile and compliance among 3810 individuals from 6 schistosomiasis endemic barangay in Palapag, Northern Samar, the Philippines. All individuals aged ≥4 were invited to participate in the study by providing stool samples for parasitological examination.

This study is part of a larger, UBS Optimus Foundation (Switzerland) funded project, where stool samples were sought from all residents ≥ 4 years across the six barangays (Fig. 1). Inclusion into this study was based on having a completed consent form and submitting at least one stool sample for analysis.

**Figure 2:**
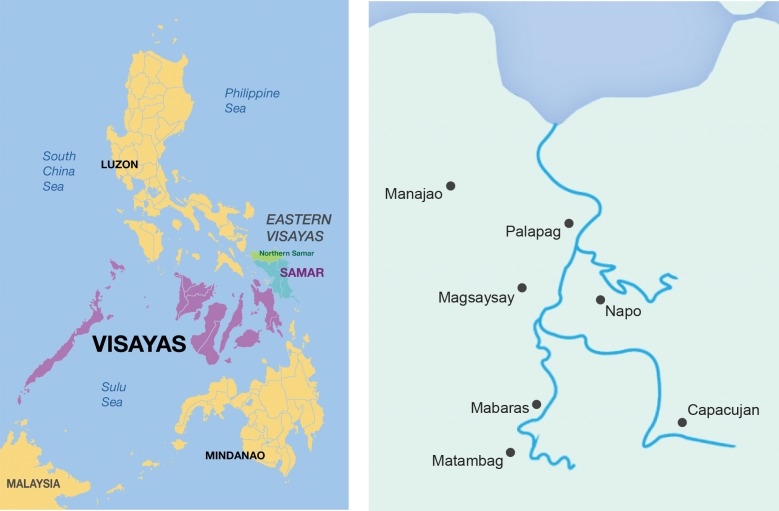
Map of the Philippines showing Northern Samar province highlighted green (Left). Map of the municipality of Palapag, showing barangay locations and rivers (Right). [48]

A cross-sectional survey was carried out in six barangays in the municipality of Palapag, Northern Samar Province, the Philippines in 2011 (Fig. 2), to determine the human *S. japonicum* prevalence and infection intensity using both microscopic (KK) and molecular (qPCR) diagnostic techniques.

Diagnostic performances of the two techniques were also assessed through sensitivity and specificity calculations.

### Study area

A barangay, the smallest administrative division in the Philippines, is the native Filipino term for a village. The study was undertaken in six barangays: Napo, Capacujan, Matambag, Mabaras, Magsaysay and Manajao, all located in the municipality of Palapag (Fig. 2). These barangays have been subjected in the past five years to annual mass treatment programs as part of the national schistosomiasis control program in the Philippines. None of the barangays selected had been treated for schistosomiasis before the onset of the study in 2011.

### Study procedures

**Sample collection**. Individual samples were obtained by handing out labelled (IDN, name, date) stool cups which were collected over seven days from study participants from each barangay. Participants submitted a stool sample between 5–10 g. In total two stool samples were sought from each individual on different days. The KK procedure (ideally performed on 2 stools, 3 slides per stool) was performed for the diagnosis of *S. japonicum*. A subset of 100 stool samples from each barangay (total = 600) was selected following KK examination for the qPCR based prevalence assessment presented here. Of the 600 samples satisfying inclusion criteria, 40 samples were excluded due to low DNA quality and quantity after DNA extraction (Fig. 1). Only 339 (60.5%) participants of the selected sub-cohort submitted two stool samples and had a complete slide set of two stools, three KK slides per stool examined. Comparison of diagnostic performance between qPCR and KK was undertaken using samples obtained from these 339 individuals such that the diagnostic sensitivity for KK was standardized across the sub-cohort.

**Kato-Katz (KK) method**. The KK was performed on all collected human stool samples. Briefly, individual stool samples were pressed through a thin gauze, the non-retained material was then used to fill a standard volume template (representing 41.7 mg of fecal material), sitting on top of a glass slide, on to which the fecal sample was transferred through the template. Cellophane soaked in glycerin-malachite green was then placed over the sample on the glass slide and pressed against a firm surface to spread the stool evenly. The slide was then viewed under a microscope after at least 30 minutes had passed. Three slides were prepared from each stool sample and the slides were read independently by a team of microscopists. Individual microscopists were blinded to the results of the other microscopists. KK was performed by microscopists either from RITM (Research Institute of Tropical Medicine) or from local schistosomiasis control units, and had previous training and experience.

**DNA extraction**. For the selected samples, 1–3 g of the remaining stool was then stored in 80% (v/v) ethanol at 4oC, for subsequent DNA isolation and molecular analysis at QIMRB, Australia. Genomic DNA was isolated from 200 mg of the stool samples using a QIAamp mini stool kit (QIAGEN) following the manufacturer’s protocol. DNA concentrations were determined using a NanoDrop 2000 (Thermo Scientific) and all DNA samples were diluted to 50 ng/μl for subsequent analysis.

**Real-time PCR**. Full details of the qPCR assay, utilizing primers which amplify a fragment of the NADH dehydrogenase I (*nad1*) mitochondrial gene, are available elsewhere [24,26,27]. Briefly, reaction mixtures of 24 μl were prepared containing 12.2 μl SYBR Green (Invitrogen), 5 pM of each primer, 7·8 μl of H2O and 2 μl of DNA (50 ng/μl) template. The PCR cycling conditions were as follows: 2 minutes initialization at 50°C, 10 minutes denaturation at 95°C, followed by 45 cycles of 15 sec denaturation at 95°C, 60 sec annealing at 60°C, 90 sec extension at 72°C and a final dissociation phase at 60–95°C. The PCR was performed using a real time thermocycler (Corbett RotorGene 6000). Melt curve analysis was performed after each qPCR. The results were quantified as eggs per gram (EPG) in the range 1–100 EPG using qPCR cycle threshold (Ct) scores [26]. Negative and positive controls were run in each qPCR. Negative controls used distilled H2O instead of template while the positive control was DNA extracted from eggs purified from the livers of infected mice.

Seeding and dilution experiments were performed to determine equivalent egg numbers (qEPG) relating to Ct scores. Eggs were purified from the livers of mice experimentally infected with *S. japonicum* [28]. Negative control human and laboratory mice stool samples were seeded with a known number of eggs and DNA extracted from these seeded samples. A standard curve using the dilutions and the results of the seeding experiments was then created to determine a range of Ct scores which corresponded to a known number of eggs. This curve was then used to quantify the qEPG in the collected human stool samples from Palapag. Based on the results of the seeding and dilution experiments a Ct of 23.0 was determined as the cut off for a positive result.

**Validation of the qPCR by microscopy**. A random selection of 20 human stool samples that were: qPCR-positive and KK-negative; qPCR- and KK-positive; or qPCR- and KK-negative were re-examined by microscopy after processing, using a modification of the formalin-ethyl acetate sedimentation-digestion (FEA-SD) technique developed recently for the precise quantification of *S. japonicum* eggs in bovine stool [26,29]. The remaining volume of stool not used for DNA extraction was used for the microscopic examination. The amount remaining varied between samples. Between 0.6–2.5 g of faeces (depending on how much of the faecal sample remained) were sieved through a 250 μm (pore opening size) nylon mesh directly onto a 38 μm nylon mesh, using water to wash the sample through the mesh and sieving until the water running out of the 38 μm mesh was clear. The remaining sediment on the 38 μm mesh was then washed into a 15 ml tube and centrifuged at 2000 rpm for 5 minutes to settle the contents. The supernatant was removed, 10% (v/v) formalin was added to a volume of 5 ml, followed by a further 5ml of 10% (w/v) potassium hydroxide solution. The tube was then vortexed thoroughly and left on a shaker at 37°C overnight. The tube was then vortexed again, the sample was centrifuged at 500 *g* for 10 min, the supernatant was removed and the pellet washed once with water. The water was removed and the final pellet resuspended in 1–2.5 ml of water, depending on the size of the pellet and the precise volume recorded. The suspension was shaken thoroughly; two 200 μl samples were spread on glass microscope slides and examined microscopically using an inverted microscope.

**Statistical analyses**. Microsoft Excel (Microsoft, Silicon Valley, LA, 2010) and SAS (SAS Institute, Cary, NC, version 9.3) software were used for data analyses. A sample was considered positive if at least one *S. japonicum* egg on any KK slide; or if a positive Ct score was seen (Ct score greater than 22.00 was considered negative, less than 21.99 was considered as positive) by qPCR. Egg counts from the KK and qEPG from the qPCR were transformed to eggs per gram and geometric mean eggs per gram (GMEPG) in infected stool samples calculated by using the log-transformed egg counts. Confidence limits were calculated using standard formulae based on the binomial distribution (prevalence) and the lognormal distribution (infection intensity). Relative diagnostic sensitivity and specificity for the KK were calculated using a subset of samples for which there was a full complement of slides (two stools, three slides per stool N = 339). Prevalence for KK was calculated from the full 560 individuals, including those with only one stool (three slides) and those with two stool (6 slides).

Relative sensitivity and specificity of the KK compared to qPCR was calculated using the qPCR as the reference standard. The qPCR was used as the reference standard as we predict this to be a better diagnostic method.

Microscope readers for the KK were blinded to the results of other readers. The qPCR was performed independently from the KK and the results compared only after the qPCR had been completed.

## Results

### Inclusion/exclusion criteria

A subset of 3810 individuals (Fig. 1) were selected for study in a larger UBS Optimus Foundation project. Of these, 69 had missing demographic data of either age or gender and were excluded. A further 331 individuals did not submit any stool samples and were also excluded (N = 3410). Of those remaining, 2005 individuals submitted two stool samples and 1405 submitted one stool sample (Fig. 1). KK was performed on each stool provided (3 slides per stool). From the remaining individuals, 600 were selected for qPCR analysis and DNA was extracted using the Qiagen kit. Of these, 40 were excluded due to low DNA quality and quantity so that the qPCR was undertaken on 560 subjects (Fig. 1). Only 339 (60.5%) participants of the selected sub-cohort submitted two stool samples and had a complete slide set of two stools, three KK slides per stool examined. Prevalence was determined on all samples from 560 individuals. Comparison of diagnostic performance between qPCR and KK was undertaken using samples obtained from these 339 individuals such that the diagnostic sensitivity for KK was standardized across the sub-cohort

### Prevalence and intensity of infection

The overall human *S. japonicum* prevalence across the six barangays in our study area in Palapag, Northern Samar, the Philippines, was considerably higher when determined by qPCR (90.2%; 95% CI 87.7–92.7) than by the KK method (22.9%; 95% CI 19.4–26.4) (Table 1). Overall infection intensity (GMEPG) was also higher when determined by qPCR (36.6; 95% CI 32.0–41.8) than by the KK (11.5; 95% CI 9.4–13.9). Males had a higher prevalence than females, although similar infection intensities were seen using both diagnostic techniques (Table 1). Barangay prevalence ranged from 84.2% (76.7–91.7) to 96.9% (93.3–100) determined by qPCR; and 18.6% (10.2–27.0) to 29.2% (19.9–38.4) by KK. Manajao had the highest prevalence of all the barangays under investigation both by qPCR (96.9%; 95%CI 93.3–100) and the KK (29.2%; 95% CI 19.9–38.4). The highest infection intensity determined by qPCR was in Napo (85.3; 95% CI 63.6–114.3); although Capacujan had the highest intensity of infection when KK was used (16.5; 95% CI 9.4–29.1).

**Table 1:** Prevalence and infection intensity (GMEPG**) of *S. japonicum* in subjects from Palapag by gender, age and barangay.

		**qPCR**			**KK**		
	**N**	**No. positive**	Prevalence % (CI*)	GMEPG**	**No. positive**	Prevalence % (CI*)	GMEPG**
**Total examined**	560	505	90.2% (87.7–92.7)	36.5 (32.0–41.7)	128	22.9% (19.4–26.4)	11.5 (9.4–13.9)
**Gender**							
Male	267	246	92.1% (88.9–95.4)	37.8 (31.2–45.8)	81	30.3% (24.8–35.9)	9.1 (6.7–12.4)
Female	293	259	88.4% (84.7–92.1)	35.4 (29.4–42.6)	47	16.0% (11.8–20.3)	13.1 (10.2–16.8)
**Age group (yrs)**							
Under 10	128	120	94.6% (89.5–98.0)	35.6 (27.3–46.5)	23	17.9% (11.2–24.7)	12.9 (8.2–20.4)
10–20	135	126	93.3% (89.1–97.6)	38.1 (29.8–48.5)	30	22.4% (16.9–27.8)	17.5 (10.6–28.8)
20–35	91	81	89.0% (82.5–95.6)	37.9 (26.4–54.4)	25	27.5% (18.1–36.8)	10.4 (6.8–15.8)
35–50	119	104	87.4% (81.3–93.5)	34.4 (25.2–47.1)	31	26.1% (18.1–34.1)	9.8 (6.7–14.5)
Over 50	87	74	85.1% (77.4–92.7)	37.1 (25.7–53.4)	19	21.8% (12.9–30.7)	7.42 (5.0–11.0)
**Barangay**							
Capacujan	96	82	85.4% (78.2–92.6)	61.7 (44.0–86.5)	23	24.0% (15.3–32.7)	16.5 (9.4–29.1)
Napo	93	84	90.3% (84.2–96.4)	85.3 (63.6–114.3)	19	20.4% (12.1–28.8)	12.4 (6.9–22.2)
Matambag	95	80	84.2% (76.7–91.7)	26.7 (20.3–35.1)	18	19.0% (16.9–27.0)	10.7 (6.1–18.5)
Mabaras	86	82	95.4% (90.8–99.9)	21.0 (16.5–26.7)	16	18.6% (10.2–27.0)	13.4 (7.1–25.2)
Manajao	96	93	96.9% (93.3–100)	24.5 (18.0–33.4)	28	29.2% (19.9–38.4)	9.3 (6.5–13.3)
Magsaysay	94	84	90.4% (84.4–96.5)	33.9 (23.0–49.7)	24	25.5% (16.6–34.5)	9.24 (6.2–13.8)

*95% Confidence Interval

** Geometric Mean Eggs per Gram of feces in infected people

### Sensitivity and specificity

The sensitivity of the KK, using qPCR as the reference standard, was 26.1% (95% CI 21.3–31.4%). Specificity was calculated as 82.8% (95% CI 64.2–94.2%).

A cross tabulation comparing KK and qPCR shows that 7 KK positive samples were negative by qPCR (Table 2). A total of 48 samples were negative by both techniques and 384 samples were negative by KK but positive by qPCR (Table 2).

**Table 2:** Cross tabulation comparison of Kato-Katz and qPCR results.

	**Positive qPCR**	**Negative qPCR**	**Total**
**Positive KK**	121	7	128
**Negative KK**	384	48	432
**Total**	505	55	560

### Cycle threshold ranges and cut offs

The range of qPCR Ct scores was compared to four categories of intensity of infection as determined by EPG (negative, low, medium and high) (Table 3). A total of 9.82% (N = 55) of samples were negative (Ct 22.00–37.21), 61.61% (N = 345, Ct 15.00–21.99) classified as low EPG (N = 123, <100 EPG), 21.96% (N = 37, Ct 12.02–14.99) medium EPG (100–400 epg) and 6.61% (Ct 7.78–11.93) as high EPG (>400 EPG) (Table 3).

**Table 3:** Association between the EPG determined by the KK and qPCR.

			**Ct-value**			**EPG determined by PCR**		
KK egg count*	N**	**qPCR pos. N**	**range**	**Median**	GM Ct^a^ (95% CI***)	**range**	**Median**	GMEPG^b^ (95% CI***)
Negative	432	384 (88.9%)	7.78–21.99	16.52	17.0 (16.6–17.3)	1.25–500	25	34.9 (29.8–40.8)
1–100	123	116 (94.3%)	9.73–21.95	16.43	16.4 (15.9–17.0)	1.25–500	25	35.3 (27.1–45.9)
100–400	5	5 (100%)	11.39–16.12	14.17	14.0 (11.9–16.6)	25–500	100	109.3 (24.5–487.2)
>400	0	N/A	N/A	N/A	N/A	N/A	N/A	N/A

^#^Range and Median of the qPCR positive samples

*KK first stool sample count only

**N of KK(Kato-Katz) samples

*** 95% Confidence interval

^a^Geometric mean eggs per gram calculated by qPCR Ct (cycle threshold)

^b^Geomeric mean eggs per gram calculated by KK

### Validation by microscopy

Twenty samples were randomly selected for further microscopic examination using a modification of the FEA-SD technique [26,29]. After processing using this procedure, *S. japonicum* eggs were found in 16 samples by microscopy (Table 4). Two of the microscopy-negative samples were also negative by qPCR and the KK, while one was positive by qPCR but negative by the KK and the fourth was positive by both techniques (Table 4).

**Table 4:** Egg recovery for microscopic validation of qPCR and comparison with the KK and qPCR.

**Sample**	**Weight (g)**	**Volume (ml)**	**EPG^a,b^**	**KK EPG^b^**	**qPCR EPG^b^**
1A	1.2	2	129.2	260	500
2A	2.43	1	6.2	0	100
3A	1.3	1	38.5	260	500
4A	1.22	2	0	0	100
5A	0.8	1	0	6.7	250
6A	0.6	1	4.2	193.3	250
7A	2	1	7.5	23.3	10
8A	1.9	1	0	0	0
9A	1.54	2	9.7	0	10
10A	1.7	2	2.9	0	250
11A	1.23	1.5	3.1	0	25
12A	1.62	2.5	19.3	6.7	50
13A	2.2	1	0	0	0
14A	1.33	2	22.6	63.3	25
15A	2.8	2	5	0	0
16A	2.1	2.5	9.22	10	25
17A	1.7	2	7.9	0	250
18A	1.8	2.5	9.7	0	25
19A	2.1	2.5	12.2	0	250
20A	2	2.5	9.4	0	10

Microscopic validation used a modified FEA-SD (Formalin ethyl-acetate sedimentation) technique applied to 20 randomly selected samples. The total weight used for the modified FEA-SD is shown in column 2. Column 3 shows the remaining volume of re-suspended sediment after processing by the modified FEA-SD. Column 4 shows the calculated EPG using the modified FEA-SD. EPG determined by the KK (Kato-Katz) and qPCR for the corresponding samples is also shown.

^a^Calculated EPG of the modified FEA-SD used for microscopic validation of qPCR

^b^Eggs Per Gram of feces.

## Discussion

In this study we found a high prevalence of schistosomiasis japonica in humans in the municipality of Palapag in Northern Samar province, the Philippines—higher than previously reported for this region. The high prevalence of *S. japonicum* mirrored the results of a small pilot study we undertook in September 2010 in Western Samar [26]. The prevalence (90.2%) and infection intensity (36.5 qGMEPG) values determined by the qPCR assay in this study were considerably higher than those obtained using the KK method (22.9%; 11.5 GMEPG). The sensitivity of the KK technique was estimated as 26.1% and the specificity as 82.8%. It is noteworthy that the KK sensitivity calculated in this study was from individuals submitting two stool samples, with three slides per stool only. The disparity between the two methods used in the current study clearly highlights the need for the development of a more sensitive technique for detecting schistosome infections in humans. The majority (N = 345, 61.61%) of qPCR positive samples were low intensity infections.

The differences in GMEPG determined by the KK and qPCR, are likely due, in part, to an overestimation of the qPCR GMEPG values. The qPCR is based on an approximation of the quantity of DNA present in one egg and how that corresponds to a Ct score. However the GMEPG obtained using the KK is also an estimate as it assumes a uniform distribution of eggs within the stool and does not take into account any egg clumping which may occur [30]. The qPCR is the more sensitive technique, especially for determining prevalence. However, there is clearly a lack of concordance with intensity of infection calculations when compared with the KK (Table 4). However samples positive by KK were only rarely negative by qPCR (Table 2). Both techniques have limitations and the methodology used to determine intensity of infection by qPCR needs to be included in future studies.

The large difference in prevalence determined using the qPCR and the KK may be explained by the fact the qPCR may be detecting DNA from adult worms or schistosomula, as well as eggs, from past or pre-patent infections. False positives due to eggs from past infections are unlikely as eggs have been shown to hatch in tissues after PZQ treatment [31]. Eggs that do not hatch due to PZQ treatment are caught in granulomas and do not hatch due to the host immune response [5]. How DNA from adult worms, living in blood vessels, would end up in the feces cannot, at present, be explained. DNA is unlikely to have originated from schistosomula as lung stage larvae would have to enter the alveolar space, be coughed up and swallowed, thereby reaching the gastrointestinal tract. If DNA from other lifecycle stages is being detected, it could account for the much higher prevalence obtained using qPCR compared with the KK. However, similar results were obtained in our pilot study in Samar for both humans and animals [26], and in an earlier study in Leyte by Wu *et al*. [27]. In humans there was a large difference in prevalence between the KK and qPCR which was mirrored in bovine infections. However, a comparison of the FEA-SD technique [29] with the qPCR indicated the *S. japonicum* prevalence in bovines determined by the two techniques was similar. This further highlights the requirement for a more sensitive microscopic technique for diagnosis of schistosomes in human fecal samples.

The qPCR is specific for *S. japonicum* DNA but to confirm and validate the qPCR results generated during this study, a random selection of fecal samples previously analysed by the qPCR were chosen and subjected to a modified version of the diagnostic FEA-SD technique, a method previously developed for the analysis of bovine fecal material [29]. We observed, by microscopy, *S. japonicum* eggs in all but two of the samples that were positive by qPCR; one of these samples was positive by the KK method. For these two samples, the total weight of stool processed was only 1.2 g and 0.8 g, respectively, which may explain why no eggs were observed after processing by the modified FEA-SD technique.

Morbidity control was implemented in China as part of the World Bank Loan Project (WBLP) (1992–2001) [32]. Recently, re-emergence of schistosomiasis japonica has been recorded in some areas where transmission control had been reported, indicating that low intensity infections can still contribute to schistosomiasis spread [16,33,34]. Additionally, it has been shown that *S. japonicum*-induced morbidity may be unrelated to intensity of infection, so that even low intensity infections can result in severe morbidity outcomes [35]. This has particular relevance when considering the KK method, which is well recognized to lack sensitivity, particularly when infection intensity is low [13,36,37]. By missing low intensity infections the burden of disease in a community may be considerably underestimated.

Transmission of *S. japonicum* is complex due to the large number of animal hosts that can act as reservoirs of infection [38]. Water buffaloes are considered the major reservoir host for schistosomiasis japonica in China [30,39–41], and this may also be the case in the Philippines where we have shown high prevalence in carabao using the same qPCR assay utilized here [26,42]. The use of this qPCR-based diagnostic is therefore not limited to detecting *S. japonicum* infections in humans and may be extremely useful as a research tool when considering transmission control of zoonotic schistosomiasis. However, qPCR has some limitations when considered as a routine diagnostic method, including the high cost of reagents and required equipment, the necessity for trained personal, and the fact it cannot be performed in the field.

Indicators for successful schistosomiasis control outlined by WHO are reduced prevalence and intensity of infection [43]. Prevalence and intensity of infection data are then used to assign categories for quantification of those suffering severe disease—assuming that a high intensity infection correlates with higher morbidity. The category of intensity infection is then used to decide on the control measures required and monitoring the control program implemented. Currently if less than 20% of a population has a high intensity of infection (>400 EPG) the area is classified as category I and screening followed by treatment is recommended. However, as previously stated *S. japonicum* morbidity can be unrelated to intensity of infection and if the KK is missing many light infections then the current guidelines for control may need to be reconsidered, particularly if the KK will continue to be used. The benefits of the KK, low cost and ease of execution, mean that it will continue to be a popular choice for control programs in the future.

Low compliance in MDA programs for schistosomiasis is an added complication to control in the Philippines. One study in Western Samar used barangay leaders to help with advocating and informing the study population about an upcoming MDA program [44]. Even with this leadership endorsement, less than 50% of individuals in the study area made themselves available for treatment. Compliance during MDA programs can be even lower through a lack of community involvement. In addition, individual case finding is rarely done, even when clear clinical symptoms of schistosomiasis are present. The focal nature of the disease, the low sensitivity of commonly used diagnostic procedures, low compliance during MDA programs and the lack of case finding make it difficult to determine the correct prevalence of schistosomiasis in the Philippines and to monitor control efforts.

## Conclusion

The results presented here clearly indicate that the prevalence of *S. japonicum* in Palapag, Northern Samar is much higher than has previously been reported, a situation that may be reflected in other endemic areas in the Philippines. Through diagnostic surveillance, molecular tools such as qPCR can provide improved assessment of the effectiveness and impact of integrated schistosomiasis control strategies.

For China, which is nearing schistosomiasis elimination [20,45], improved diagnostic techniques such as real-time qPCR and conventional PCR (cPCR), will be required to monitor low intensity infections. Current guidelines for schistosomiasis control that are based on prevalence and intensity measurements obtained by the KK may also need to be revised as improved sensitive diagnostic techniques, including the qPCR, become more extensively used. The WHO recommends mass treatment, particularly of school aged children, as a way to prevent morbidity in later life [46]. However with zoonotic transmission occurring, simply treating human cases will not result in elimination of *S. japonicum*. Integrated control, utilizing mass treatment in combination with other strategies to break the life cycle of the disease, has been suggested as a more cost-effective way to achieve elimination [47]. The advent of more sensitive diagnostics, such as qPCR, may assist in achieving elimination in the long term.

## Supporting Information

S1 Checklist STARD Checklist. (DOCX)
